# Genetic and Morphometric Variability of *Triatoma sordida* (Hemiptera: Reduviidae) from the Eastern and Western Regions of Paraguay

**DOI:** 10.3389/fpubh.2014.00149

**Published:** 2014-09-19

**Authors:** Nilsa E. Gonzalez-Britez, Hernán J. Carrasco, Clara Elena Martínez Purroy, M. Dora Feliciangeli, Marisel Maldonado, Elsa López, Maikell J. Segovia, Antonieta Rojas de Arias

**Affiliations:** ^1^Departamento de Medicina Tropical, Instituto de Investigaciones en Ciencias de la Salud (IICS), Universidad Nacional de Asunción, Asunción, Paraguay; ^2^Laboratorio de Biología Molecular de Protozoarios, Facultad de Medicina, Instituto de Medicina Tropical, Universidad Central de Venezuela, Caracas, Venezuela; ^3^Instituto de Investigaciones Biomédicas (BIOMED), Universidad de Carabobo, Maracay, Venezuela; ^4^Centro para el Desarrollo de la Investigación Científica (CEDIC)/Díaz Gill Medicina Laboratorial/Fundación Moisés Bertoni, Asunción, Paraguay

**Keywords:** *Triatoma sordida*, Chagas disease, RAPD, morphometric analysis, feeding content

## Abstract

*Triatoma sordida* is widely distributed throughout the Chaco and the Eastern Region of Paraguay. It is associated to palm trees and artificial ecotopes located in peridomestic environments. The aim of this work was to determine genetic and morphometric variability and feeding behavior among population of *T. sordida* captured in domicile and peridomicile areas of Paraguay. Feeding contents and levels of genetic and morphometric variation were determined in 124 *T. sordida* from domicile and peridomicile populations of San Pedro and Paraguarí departments of the Eastern Region and Boquerón and Presidente Hayes departments of the Western region using Double Diffusion Gel, random amplified polymorphic DNA (RAPD), and head and wings morphometry. Morphometric analysis revealed isolation of populations by geographic region and larger size in triatomine populations from the Western Region. RAPD showed no specific patterns for domicile and peridomicile populations. The estimator of diversity (*F*_ST_; 0.08) and high gene flow obtained (*N*_m_; 5.7) did not allow the establishment of genetic differentiation within the same region. The blood meal source showed that poultry feeding was 38% of host preferences, and human blood was the second feeding preference (24%) in the insects from the Eastern Region while poultry feeding was predominant in those from the Western Region (30%). This work showed homogeneity between *T. sordida* populations of the same region and between domicile and peridomicile. The genetic diversity was determined among *T. sordida* populations of both geographical regions suggesting differentiation associated to eco-geographical isolation by distance. It is important to notice that pattern feedings were different between the two regions. Further studies should be focused on how phenetic and genetic variations could be related to the adaptation capacity of these triatomine populations to domicile, increasing their vector potentiality in the transmission of Chagas disease.

## Introduction

The subfamily Triatominae (Hemiptera: Reduviidae) includes over 144 species of strictly hematophagous insects, considered potential vectors of *Trypanosoma cruzi* among mammals. However, not all of them are epidemiologically important (1–4). In Paraguay, 11 species of triatomines have been registered and from them *Triatoma infestans* (5) and *T. sordida* (6) have been found naturally infected with *T. cruzi* (7, 8).

In the Southern Cone countries, the most important hematophagous vector involved in the transmission of Chagas disease is *T. infestans*. *T. sordida* of wild origin seems to have been disseminated from Brazilian plateaus toward the south, and now is found in Argentina, Bolivia, Paraguay, and Uruguay where it occupies extensive geographical areas but generally in small populations of individuals (9, 10). *T. sordida* is considered as a ubiquitous species with high ecological potential living in various ecotopes and feeding from different sources. This insect could withstand large environmental changes that cause the disappearance of his competitors and could widen its ecotopes to dead and dry trees (11). However, these ecotopes usually do not offer feeding sources, stimulating its dispersion to peridomiciles and domiciles and there is ever-greater contact inside and around houses with species other than *T. infestans* that were not very important for vector transmission in the past because they used to be found only in natural ecotopes, as *Triatoma sordida*. Their epidemiological importance regarding vector transmission of Chagas disease is still low, but they may become a bigger problem if they become domesticated, thereby occupying the empty place left by *T. infestans* (12, 13).

On the other hand, the sympatry with *T. infestans* in domicile and peridomicile is known as well as the diversity of the ecotopes it occupies and the difficulty this has meant for its control. *T. sordida* is associated with re-infestation sources of dwellings treated with insecticides and currently is considered a potential vector of Chagas disease (11, 14–16).

Morphometric and molecular analyses are important tools that provide evidence of the population structure of insect vectors. Enzymatic and genetic studies performed on this triatomine species have confirmed the variability of loci in two groups (17–19). *T. sordida* group 2 seems to be restricted to the Chaco and group 1 is widely distributed in Bolivia and Brazil (18, 20). Besides, the genetic distances between both populations led to infer the hypothesis of recent cryptic speciation (21).

The study based on morphometric analysis and the molecular patterns of random amplified polymorphic DNA (RAPD) in *T. brasiliensis* have reported the existence of a common relationship between wild and domiciliary populations (22). Similarity, the gene-flow index and reduced genetic divergence found between different populations of *Triatoma rubida* support sub-specific designation for this species (23). In relation to *T. sordida*, low levels of genetic variation among populations of southeastern Brazil have been reported through the analysis of 28 allozyme loci. None of these loci presented significant differences between any pair of populations, and only two showed polymorphism, accounting for low levels of heterozygosity (10). Similarly, the genetic study of *T. sordida* from different ecotopes of Paraguay revealed low genetic diversity levels suggesting that extra-household populations could represent an important epidemiological link to maintain the transmission of trypanosomatids (24).

Thus, triatomines studies based on diverse molecular markers have been used to clarify phylogenetic and evolutionary relationships between species, apart from inferring divergences and population structure, as it has been demonstrated previously in others triatomines, where wide polymorphism between discrete populations suggest the existence of a species complex (25, 26). These facts suggest an increment of the epidemiological significance of vectors considered secondary (12, 22). In the case of *T. sordida*, the polymorphism levels and its implication in the infestation of dwellings in endemic areas for Chagas disease in Paraguay are still unknown. The objective of the present work was to determine the feeding behavior and genetic and morphometric variability among population of *T. sordida* captured in domicile and peridomicile of the two geographical regions of Paraguay. Finally, this study contributes to improve surveillance strategies embracing this potential vector.

## Materials and Methods

### Study sites, bug collection, and parasitological search

The Eastern Region is humid, sub-tropical, composed by valleys, small hills, and wooded areas. The average annual temperature is 24.3°C and the average annual rainfall is between 1000 and 1600 mm. (27). The Western or Chaco Region is characterized by extreme temperatures ranging from 45°C in spring and summer to 27°C in winter with annual minimum rainfall of 100–900 mm. (28). Both regions are separated by an important ecological barrier, the Paraguay River.

**Figure 1:**
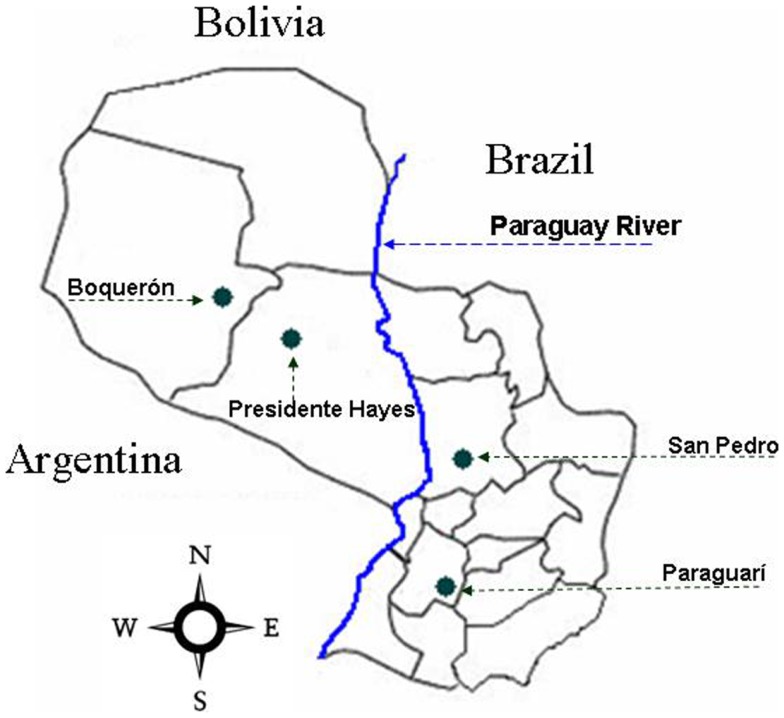
**Geographical localization of the study area**. The green dots show the capture places in the different departments of the Republic of Paraguay. Departments: Boquerón (BO), Presidente Hayes (PH), Paraguarí (PA), and San Pedro.

The specimens were collected by manual capture in poultry house, stables, and pigsties of peridomicile and intra-domicile areas of San Pedro (SP) and Paraguarí (PA) departments of the Eastern Region of Paraguay; Boquerón (BO) and Presidente Hayes (PH) departments of the Western Region (Figure 1). All triatomines were maintained alive and classified previously as *T. sordida* according to Lent and Wygodzinsky (29).

One hundred twenty-four specimens were analyzed: 63 males and 61 females (Table 1). All insects were studied by morphometric analysis and half of them were studied by molecular methods. Parasitological search was also carried out microscopically in all insects by the direct observation of their feces and morphological identification performed after staining with Giemsa at 400× in an Olympus microscope in order to identify trypanosomatids. Characteristic morphological features of *T. cruzi* were identified as described by Hoare (30).

**Table 1:** **Distribution of *T. sordida* captured in different localities of the Paraguayan regions**.

Localities	Paraguayan region	Latitude	Longitude	Number of *T. sordida* evaluated		
				Males	Females	Total
Cerro Guy (PA)	Eastern	25°41′39.95″	57°10′51.96″	16	20	36
San Pedro (SP)		24°05′19.39″	57°04′35.47″	15	11	26
Gral. Bruguez (PH)		24°45′13.31″	58°49′37.29″	17	16	33
Galilea (BO)	Western	22°35′00″	59°55′59.90″	15	14	29
				63	61	124

*Departments: Boquerón (BO), Presidente Hayes (PH), Paraguarí (PA), and San Pedro (SP)*.

### Morphometric analyses

**Figure 2:**
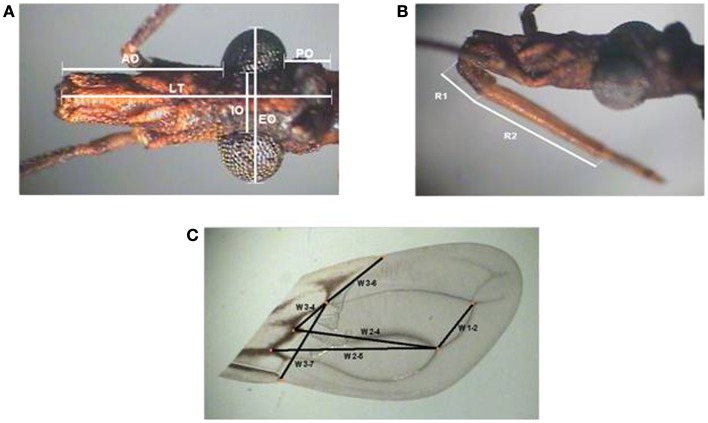
**Diagram of head and wings measurements used for the morphometry of *Triatoma sordida* is shown**. **(A)** Dorsal (left) and **(B)** lateral (right) views of the head indicating morphometric measurements taken. AO, anteocular distance; PO, post-ocular distance; LT, total length; IO, inner distance between eyes; EO, outer distance between eyes; R1, length of first rostral segment; R2, length of second rostral segment. **(C)** Dorsal view of right wing with measurements taken for the distance between the following points: W1–2 between point 1 and 2, W2–4 between point 2 and 4, W2–5 between point 2 and 5, W3–6 between points 3 and 6, W3–4 between points 3 and 4, and W3–7 between points 3 and 7.

Head and wings were selected according to protocols previously described (31–33). In the head, seven homologous points were selected (Figures 2A,B), while six distances measurements between the points of intersection of the veins were used for wings (Figure 2C). All measurements were made in duplicate by the same researcher and the images were captured using a lucid camera connected to an Olympus stereoscopic microscope DF Plan 1×.

The matrixes were tabulated by sex and population. The sexual dimorphism and Guillaumin profile were determined to obtain information about the general size of a group respect to other (34, 35). The principal components analysis (PCA) was carried out using covariance matrix from which a factorial map was constructed to show the differences in size and shape among sexes and populations (36). The free-allometry analysis for shape differences was performed after the discriminant analysis (DA) made on the set of common principal components (CPC), excepting the first common principal component (CPC1), according to a protocol described by Dujardin et al. (37). For this, it was indispensable to check the compatibility with the model of CPC using a Chi-square goodness-of-fit test (*X*^2^). All parameters were calculated using the JMP 4.0.0 (38) and NTSYSp.c version 2.10p (39) statistical packages.

### Feeding source analyses

#### Extraction of blood content

The intestinal content of 62 adult specimens (29 males and 33 females) was extracted; 34 of them were from the Eastern Region (PA) and 28 from the Western Region (Pte. Hayes). In order to do this, a section was made in the front third of the abdomen of the specimens. When the content volume was insufficient, the complete promesenteron was transferred to a vial (40). Each vial had the same blood sample with 180 μL of 4% saline solution and 20 μL of 10% crystal violet and the mixture was maintained at 4°C for 24 h (41).

#### Determination of the feeding source

The Gel Double Diffusion method was carried out in a glass slide (7.5 cm × 5 cm) using 3.5 mL of 1.3% agar (I.D. Oxoid Agar) diluted in veronal hydrochloride buffer (pH 8.6). This preparation was maintained in a humid chamber for 24 h (40, 42). In the agar, there was a central hole that was filled up with the specific antiserum and six peripheral holes, five containing the diluted antigen (blood sample from different triatomines) and one with saline solution (negative control).

The antisera used for the identification of the feeding source was against human, poultry (chicken), dog, cat, goat, mice, and guinea pig blood and were also put in contact with the intestinal content of the triatomines searching for the corresponding antigen. All the antisera were prepared and tested previously in the Laboratory of General Ecology of the University of Buenos Aires, Argentina.

### Random amplified polymorphic DNA

The extraction of DNA was carried out according to the protocol of Promega Wizard Genomic Purification Kit, USA (43) in five legs of each insect (25).

The amplification reaction was performed according to the protocol of Williams et al. (44) modified by Carrasco et al. (45). The DNA of 62 specimens: PH (8 males, 8 females), BO (7 males, 8 females), PA (8 males, 8 females), SP (8 males, 7 females) was amplified with four primers to distinguish triatomine species of the same genus or identify affinities between species. The primers were: A_1_ (5′-TCACGATGCA-3′), A_2_ (5′-GAAACGGGTG-3′), L_4_ (5′-GTGGATGCGA-3′), and L_5_ (5′-AAGAGCCCGT-3′). The PCR was set up as follows: a final volume of 25 μL PCR mixture that contained 0.25 mM dNTPs (Pharmacy Biotechnology, Sweden), 10 pmol of primers, 1.0 unit of *Taq* polymerase (Gibco Life Technology), 5 ng of DNA template in a buffer with 2 mM MgCl; 50 mM KCl, 10 mM Tris–HCl, pH 8.8 was used; each amplification included a DNA-free negative control. The visualization of the products was obtained using 2.5% ultra-pure agarose gel electrophoresis stained with ethidium bromide. The bands obtained were digitalized by KODAK 1D (Kodak Digital Science) software.

### Data analysis

The binary matrix was built using the specimens that generated better band reproducibility and intensity. For the analysis, it was assumed that the *T. sordida* populations were in Hardy–Weinberg equilibrium and that there were no selection processes favoring any particular genotype. All loci were entered in a binary matrix and a similarity index was obtained from this matrix (46) in order to build a UPGMA (unweighted pair group method of arithmetic mean) dendogram. The genetic distance was based on Nei (47) and the index of genetic differentiation (*F*_ST_) was determined according to Nei and Chakraborty (48), which is used to examine the level of genetic divergence among sub-populations and provides an estimation of the genetic flow (*N*_m_). These parameters were analyzed using the software POPGENE (version 1.31) (49).

## Results

### Parasitological assays

Feces of 124 insects were analyzed looking for *T. cruzi*. None of them showed natural *T. cruzi* infection, confirming in this occasion the low infection rates of *T. sordida* specimens in both regions (data not shown).

### Morphometric assays

#### Size analysis

The Guillaumin profile allowed, in general, determining that individuals from the Western Region were larger than those from the Eastern Region. The sexual dimorphism of the analyzed structures (W2–5, W3–6, W3–7, W2–4, W1–2, W3–4, AO, R_2_, R_1_, EO) was significant (*p* = 0.01–0.0001), excepting the post-ocular (PO) distance of the head (*p* = 0.08). The Bonferroni correction showed that the characters of the wings differentiated the sexual dimorphism better than those of the head.

**Figure 3:**
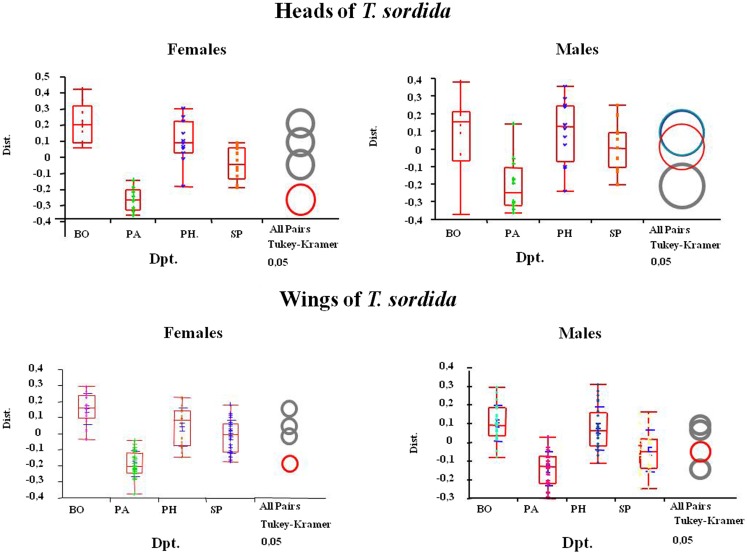
**Size differences between geographical populations (from the indicated departments) in heads (up) and wings (below) of *T. sordida* males and females are shown**. The arithmetic mean can be observed as a horizontal line that divides the boxes in two. The ends of the boxes correspond to 25th and 75th quantiles while the vertical lines show the maximum and minimum values of the distribution. BO, Boqueron Department; PA, Paraguari Department; PH, Presidente Hayes department; SP: San Pedro Department.

The size analysis using the principal components showed that, although females were consistently larger than males, the populations from BO department had significantly larger wings and heads than those from the other studied localities (Figure 3). The significance of the size differences among insects of different departments was determined by Kruskal–Wallis non-parametric ANOVA test, using the mean of each group separately (*p* = 0.0001) for head and (*p* = 0.001) for wings.

#### Conformation and shape analysis

**Figure 4:**
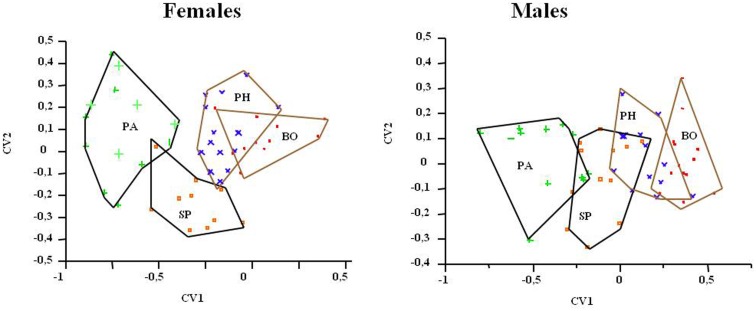
**Discriminant analysis (allometry-free), derivate of variables of *T. sordida* heads in four different populations**. The polygons contain the specimens of each *T. sordida* population. BO, Boquerón Department; PA, Paraguarí Department; PH, Presidente Hayes Department; SP, San Pedro Department.

The DA for isometry-free variables evidenced the significant separation of the triatomine populations of the two geographical regions, better reflected by females according to the values of Wilks lambda = 036, *p* = 0.0003 (head) and Wilks lambda = 042, *p* = 0.0001 (wings). With the elimination of the allometric size, only the head variables were compatible with the CPC (common principal component) model (*x*: 33.57, *p* = 0.2982 in females and *x*: 36.29, *p* = 0.1987 in males). Through the DA, the specimens were correctly classified into their respective groups with considerable concordance (Kappa between 0.66 and 0.83). This canonical variation analysis also showed the isolation of PA population for both sexes (Figure 4).

### Feeding source analyses

**Figure 5:**
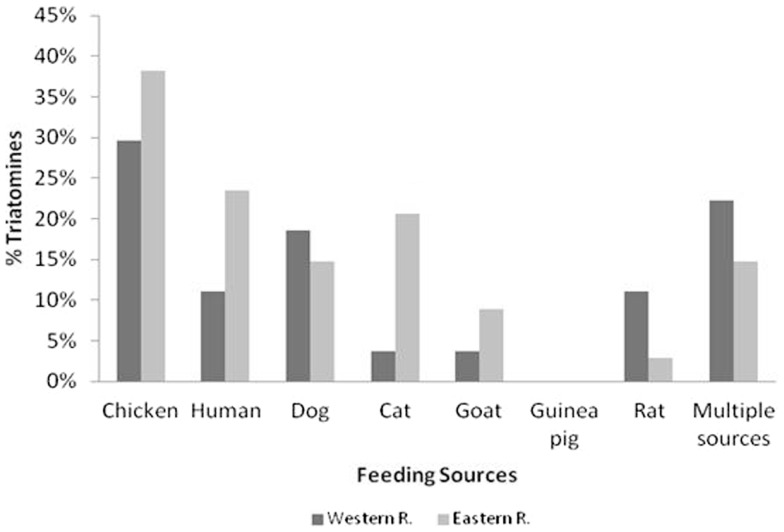
**Frequency of blood ingestion for *T. sordida* in the Eastern and Western Regions is shown**. The results of most common vertebrates are included. In the category “Multiple Sources,” the specimens with blood ingestion from several vertebrates are included.

Figure 5 shows the percentages of *T. sordida* intestinal content that reacted with different vertebrate hosts. The preferred feeding source was varied and included the finding of blood from pets, poultry, and even rodents. The most frequent feeding source of the specimens collected inside and around the houses was poultry blood (hen or chicken): 30% for Gral; Bruguez community of Western Region and 38% for Cerro Guy community of Eastern Region. In the latter, the most frequent second blood source was human (24%) followed by cat and dog blood. In the Western Region, the second frequent blood source was multiple blood (feeding on several animals) where the most common blood mixtures were poultry-human, poultry-rat, and poultry-dog-cat.

### Molecular analysis

**Figure 6:**
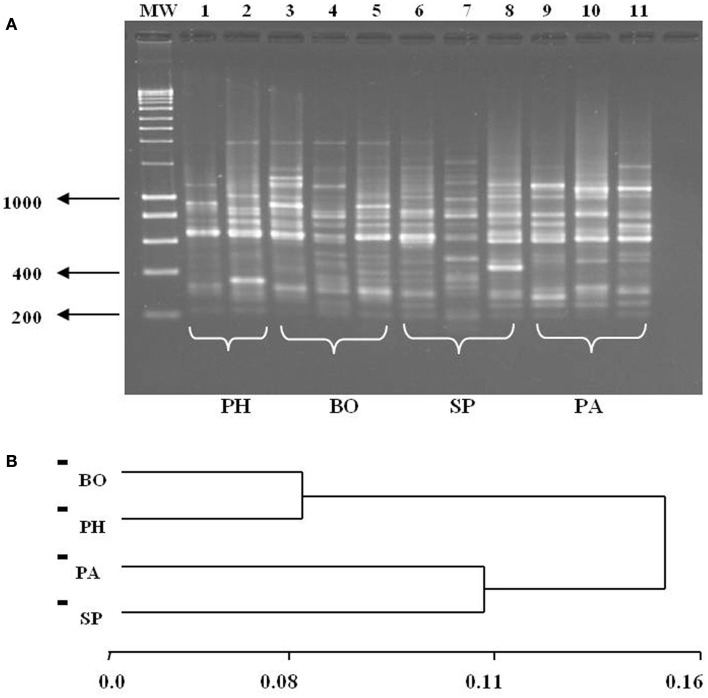
**(A)** RAPD profiles with DNA extracted from different *T. sordida* populations using the primer A2. Lanes 1–2: Pte. Hayes (PH), Lanes 3–5: Boquerón (BO), Lanes 6–8: San Pedro (SP), Lanes 9–11: Paraguarí (PA), CN, negative control. PM, Hyperladder I Bioline. **(B)** Dendrogram of grouping four *T. sordida* populations by genetic distance based on Nei’s genetic distance. BO, Boquerón; PH, Pte. Hayes; PA, Paraguarí; SP, San Pedro.

The RAPD profiles were complex (Figure 6A), and the size variation of amplified fragments ranged from 200 to 2500 bp. A total of 98 polymorphic loci generated by four primers were selected for comparative analysis according to their intensity, resolution, and reproducibility. The remarkable polymorphism showed patterns of different bands, but specific patterns were neither observed for insects collected in intra-domicile/peridomicile environments nor for the different departments. The similarity matrix was calculated in accordance with the degree of paired band between each pair of individuals (46) from which the UPGMA tree was generated (Figure 6B). On the other hand, the grouping of the means of allelic frequency showed two particular groups, corresponding to specimens from Western (BO and PH) and Eastern (SP and PA) Regions. The Nei’s genetic distance (1978) indicated a larger separation between the populations separated by larger geographic distance (approximately 520 km), i.e., between BO and PA. The analysis with the POPGENE software showed an estimate of the genetic diversity value (Gst) of 0.08 while the migration index value (*N*_m_) was 5.7 individuals per generation.

## Discussion

This is the first report of *T. sordida* invading and trying to colonize houses in both Paraguayan regions. These areas have been intensively sprayed and a tendency of invasion has been observed when residual insecticide activity ends. Previous reports demonstrated very low rates of domiciliary colonization of *T. sordida* in Argentina and Brazil (11, 15), but the scenario is quite different in some areas of Bolivia (50).

We have explored the intraspecific relationships among *T. sordida* populations from different endemic areas for Chagas disease in Paraguay, considering the limited information on their behavior and based on reports about their wide dispersion and high peridomicile infestation (51). A previous study referred that human blood is the second more important feeding source of *T. sordida* in endemic areas of Paraguay (52), which suggests an increment in the transmission risk of the parasite without the necessity of establishing colonies in rural dwellings. In this study, we still observed human blood as the second feeding source in *T. sordida* from the Eastern Region but insects captured at the Western Region mainly showed a peridomestic pattern feeding where blood from animals, including sylvatic ones, was detected. Although triatomines were not found positive for *T. cruzi* infection, this new scenario should be taken into consideration in locations where *T. sordida* is frequently found inside the houses where *T. infestans* is absent.

Both morphometric and molecular analyses were carried out to determine the genetic structure of triatomines in order to generate useful information to establish more effective strategies for vector surveillance, incorporating information on their phenotypical variations and sexual dimorphism, excluding changes caused by environmental factors (53) that were corroborated by RAPDs techniques.

Our study did not show any differences in the sexual dimorphism of specimens from peridomicile and domicile, suggesting a continuous exchange with the sylvatic triatomine populations and no transition from sylvatic to domestic habitats or a domiciliation process and adaptation to new habitats were found as it was demonstrated for *T. infestans* and *Panstrongylus geniculatus* (53–55). That is to say that in spite of the frequent finding of *T. sordida* species in the domicile, specific changes related to domiciliation process were not observed, suggesting their temporary presence in dwellings, which was already reported by other authors (56). According to Jaramillo et al. (57), the size of triatomines can be modified in response to environmental changes, thus the significant variation shown by the multivariate analysis of the isometric size in triatomines from Western and Eastern Regions corroborates the influence of micro-environmental conditions, like fourrage disposition in peridomiciles, which leads to the permanency of the insects in such ecotopes. The triatomines from PA department presented significant morphometric variations in relation to the other populations, suggesting a recent adaptation to peridomiciliary ecotopes. Although Chaco populations have conditions for colonization, the triatomines remain in sylvatic ecotopes that are more unstable (58–60). On the other hand, the decrease of size in PA can be attributed to the less favorable modified environments due to the big density of insects and the competition for the nutritional source available in that ecotope (61). The migration of triatomines to peridomicile is produced in response to agricultural habits and destruction of natural forests for anthropic action, even more if we consider that the PA department has been subjected to frequent modifications (agricultural area) or faced control interventions with insecticides. This leads to the dispersion of triatomines and the later adaptation to “new ecotopes”; or simply this adaptation is compatible with the hypothesis of restricted migrations if such ecotopes have enough food sources available (7, 62). According to Dujardin et al. (61), this adaptation to different ecotopes (ecological pressure) is the main mechanism that drives the speciation in the sub family Triatominae. It is important to notice that Chagas Disease Control Program in Paraguay recently showed more frequent domiciliary infestation of *T. sordida* in several localities of the PA department during the monitoring man/hour search carried out by its technical personnel.

With the elimination of the allometric changes, the DA evidenced the separation of the type morphologies of each region, which can be associated with environmental differences, geographical distances, or the intervention of genetic factors. The significant discrimination observed between the populations of PA and BO suggests a separation due to the distance among both in agreement with what was reported in a previous study made with sensilla patterns between populations of *R. prolixus* of the Andean area and oriental plains of Colombia (63). On the other hand, the observed overlapping of the factorial map among populations of the same region leads us to think of a process of passive migration.

The genetic variability can be a consequence of the metric differences observed in specimens from different habitats, i.e., that the metric characteristics are almost exclusively under environmental control and the genetic variations could be the result of the contribution of genetic and environmental features (64). The RAPD method showed genetic structuring between *T. sordida* populations of both geographical regions and the genetic similarity was bigger among populations of the same region, suggesting the existence of a constant gene flow among them. This seems logical but such grouping may be reflecting recent events with few codon changes caused by the adaptation process of sylvatic populations to artificial ecotopes.

The similarity analysis shown by the *F*_ST_ index suggests an exchange among insect populations from neighboring departments that gets reduced among regions. Therefore, the *F*_ST_ estimator shows little genetic differentiation and according to Nei’s classification (1973), this fact seems logical as they are insects of the same species. However, we suppose that the separation between regions and the morphobiometric differences of PA populations could be related to the genetic changes caused by local selective pressures. The grouping observed in the dendrogram could be associated with epidemiological differences in their respective origin focuses, considering that the Western Region is an area with high pressure of triatomine infestation (16, 58). However, to confirm this we suggest increasing the study of *T. sordida* populations in Paraguay and the use of more sensitive molecular markers to compare these findings with cryptic speciation groups previously described (18, 34).

The dendrogram is similar to the result previously obtained for *T. infestans* populations that demonstrated allelic differences among neighboring localities, which increase among populations more distant from each other (65). It has been suggested that the genetic isolation by geographical distance greatly contributes to the genetic variability of triatomine populations caused by the passive dispersal of the insects in association with human migrations, resulting in the founder effects and subsequent genetic drift (33). The migration index obtained in this study suggests the mobility of *T. sordida* between neighboring populations and the results presented suggest a genetic homogeneity between *T. sordida* from the same region, which is due to the permanent genetic flow between neighboring populations. However, the observed heterogeneity between specimens from Western and Eastern Regions could be associated with the big distances and even with the presence of the Paraguay River as a geographical barrier, which would be in agreement with the separation obtained with the morphometric analyses, i.e., that the differences between PA and BO involves a differentiation process, possibly associated with the eco-geographic isolation by distance and absence of genetic flow. Feeding behavior also confirm differences in these two populations, while triatomines from the Western Regions showed a poultry feeding profile, patterns of the insects from Eastern regions were associated with poultry feeding and human blood feeding profiles in triatomines captured in domicile. Mixed feeding showed an intense mobilization behavior of these triatomines between peridomicile and domicile areas.

It is important to notice that the genetic analysis has shown intraspecific divergences that allow us to think of possible gene variations involved in the shape expression, although we could not discard the possibility that the separation reflected in the dendrogram is influenced by the variation of allelic frequencies of the individuals. In this case, new questions arise and further studies will be required with models of population genetics to obtain better markers related to the infestation risk of potentials triatomines, mainly as a consequence of the control of *T. infestans* in Paraguay as other secondary vector species such as *T. sordida* are more frequently detected in the studied region.

Further studies should be focused on how phenetic and genetic variations could be related to the adaptation capacity of these triatomine populations to domicile, increasing their vector potentiality in the transmission of Chagas disease.

## Conflict of Interest Statement

The authors declare that the research was conducted in the absence of any commercial or financial relationships that could be construed as a potential conflict of interest.
